# Geroscience in heart failure: the search for therapeutic targets in the shared pathobiology of human aging and heart failure

**DOI:** 10.20517/jca.2024.15

**Published:** 2025-01-14

**Authors:** Claire Castro, Constance Delwarde, Yanxi Shi, Jason Roh

**Affiliations:** 1 Cardiovascular Research Center, Massachusetts General Hospital, Boston, MA 02114, USA.; 2 Center for Interdisciplinary Cardiovascular Sciences, Brigham and Women’s Hospital, Boston, MA 02115, USA.; 3 Harvard Medical School, Boston, MA 02115, USA.

**Keywords:** Translational research, heart failure, genomics, proteomics, aging biology, geroscience

## Abstract

Age is a major risk factor for heart failure, but one that has been historically viewed as non-modifiable. Emerging evidence suggests that the biology of aging is malleable, and can potentially be intervened upon to treat age-associated chronic diseases, such as heart failure. While aging biology represents a new frontier for therapeutic target discovery in heart failure, the challenges of translating Geroscience research to the clinic are multifold. In this review, we propose a strategy that prioritizes initial target discovery in human biology. We review the rationale for starting with human omics, which has generated important insights into the shared (patho)biology of human aging and heart failure. We then discuss how this knowledge can be leveraged to identify the mechanisms of aging biology most relevant to heart failure. Lastly, we provide examples of how this human-first Geroscience approach, when paired with rigorous functional assessments in preclinical models, is leading to early-stage clinical development of gerotherapeutic approaches for heart failure.

## Graphical Abstract

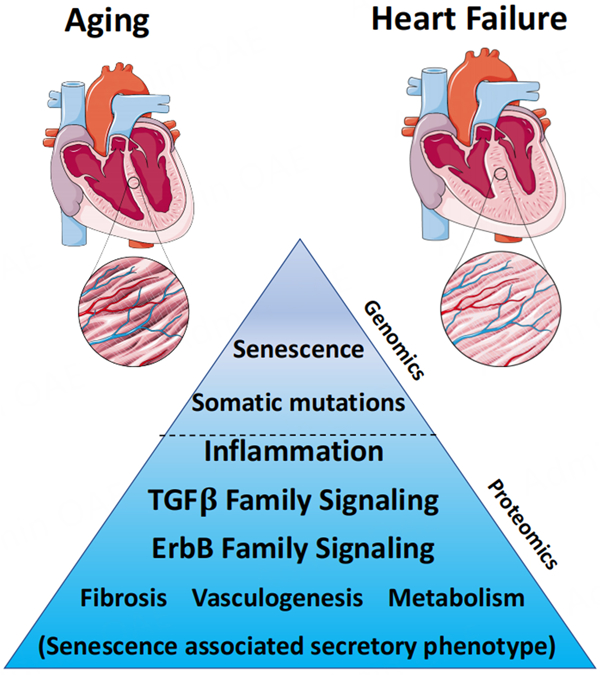

## INTRODUCTION

### Aging in heart failure epidemiology

Heart failure (HF) is a clinical syndrome defined by symptoms or signs of pulmonary or systemic congestion caused by a structural and/or functional cardiac abnormality^[1]^. Approximately 64 million people worldwide are currently living with HF, which has more than doubled in the past two decades^[2,3]^. While multiple factors, including rising rates of cardiometabolic disease (i.e., obesity, diabetes), are contributing to the increasing prevalence of HF, a major impetus for the rising number of HF cases is the progressive aging of the world’s population. Currently, ∼14% of the world’s population is > 60 years old; by 2050, this figure is anticipated to reach 22%, representing more than 2 billion older adults worldwide (https://who.int/news-room/fact-sheets/detail/ageing-and-health).

Similar to its role in most chronic diseases, age is a major risk factor for HF. The incidence of HF is over 4-fold higher in adults aged > 65 years compared to their younger counterparts^[4]^. Not surprisingly, adults aged >70 years account for ∼2/3 of the world’s HF cases^[2,3]^. Despite advancements in the treatment of HF and its associated risk factors, the prognosis for older adults with HF, whether it be heart failure with reduced ejection fraction (HFrEF) or the increasingly more common heart failure with preserved ejection fraction (HFpEF), remains poor. HF continues to be a leading cause of hospitalization among older adults^[2,3]^. Moreover, after an index HF hospitalization, nearly 2/3 of these older adults will be re-hospitalized and ∼1/3 will be dead within one year^[3,5]^. These outcomes highlight the unmet need for effective preventative and therapeutic strategies for HF.

### Aging biology: a new target for therapeutic development in heart failure?

The search for therapeutic targets for HF has evolved with our understanding of its pathophysiology. The recognition that chronic activation of compensatory neurohormonal pathways (i.e., adrenergic nervous system, renin-angiotensin-aldosterone [RAAS] system) contributes to maladaptive myocardial remodeling and HF progression revolutionized drug development for HFrEF from the 1990-2000s^[6]^. In the past two decades, insights into the mechanistic underpinnings of various subtypes of HF, including cardiac amyloidosis and hypertrophic cardiomyopathy, have led to the recent approval of targeted therapeutics (e.g., tafamidis, mavacamten) for these specific forms of HF^[7,8]^. More recently, sodium-glucose cotransporter 2 (SGLT2) inhibitors and glucagon-like peptide 1 (GLP1) agonists have emerged as effective therapies for both HFrEF and HFpEF^[9–11]^. While the mechanisms mediating their benefits in HF are multifactorial and not yet fully understood, their effects on metabolic remodeling have invigorated efforts to target this pathobiology in HF.

Conversely, age has long been a thorn in HF management. Conventional thinking construes age as an important risk factor to recognize and consider in the management of older adults with HF, but one that cannot be modified. This concept, however, is changing with the rapidly evolving field of Geroscience^[12–14]^. Geroscience is the study of the biology of aging that prioritizes human translation. It is based on a central hypothesis that aging is a primary cause of most chronic diseases in older adults, and thus, attenuating or reversing biological processes that govern aging could simultaneously delay or treat many of these diseases, including HF^[12,15]^. This hypothesis is, of course, dependent not only on aging being truly malleable, but also on the establishment of shared causal biology between aging and disease. If these two criteria are met, then simply put, age becomes more than just a risk factor, but one that can be targeted for therapeutic development.

**Figure 1.**
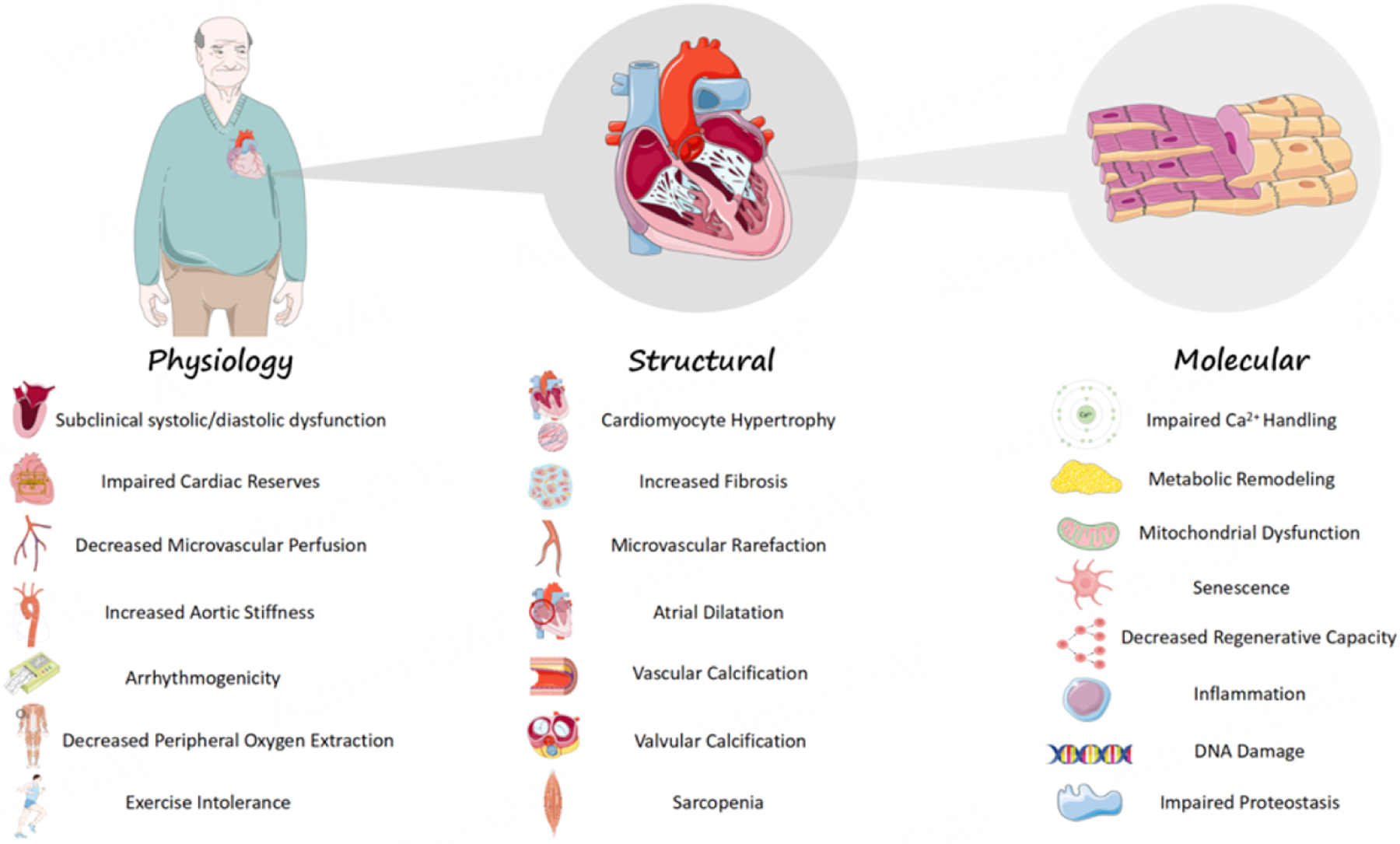
Schematic of the physiological, structural, and molecular changes that occur with cardiovascular aging. This illustration was partly generated using images adapted from BioRender and Servier Medical Art (https://smart.servier.com). Servier Medical Art is provided by Servier, licensed under a Creative Commons Attribution 4.0 license (https://creativecommons.org/licenses/by/4.0/).

While the field of Geroscience is relatively new, the hypothesis that cardiovascular aging is part of the causal pathophysiology of HF has been around since at least the 1940s^[16]^. Traditional risk factors (e.g., hypertension, diabetes, coronary artery disease, *etc*.) account for only ∼53% of the population attributable risk for HF in the elderly^[17]^, suggesting that intrinsic elements of aging may be unrecognized drivers of HF in this population. Indeed, the aging cardiovascular system exhibits numerous parallels to HF, from the physiological to the molecular level [Figure 1]. Elegant physiology studies in healthy humans, across the spectrum of age, have shown that normal aging is associated with progressive impairments in subclinical systolic and diastolic function, cardiac reserves, peripheral oxygen extraction, and overall exercise capacity, along with changes in macro- and microvascular function^[18–21]^. At a cellular and molecular level, aging is associated with cardiomyocyte hypertrophy, impaired Ca^2+^ cycling, mitochondrial dysfunction, altered proteostasis, dysregulated metabolism, loss of regenerative potential, in addition to endothelial dysfunction and increased fibrosis. A detailed discussion of these changes is beyond the scope of this paper, but they have been extensively reviewed in recent publications^[13,14,21–23]^. Notably, while cardiovascular aging, in itself, does not equate to clinical HF, it results in a substrate in older adults that can easily transition into the disease manifestation of HF. The question is no longer whether the aged cardiovascular system is more susceptible to HF, but more so, what biological mechanisms of aging drive this increased HF vulnerability in older adults.

**Figure 2.**
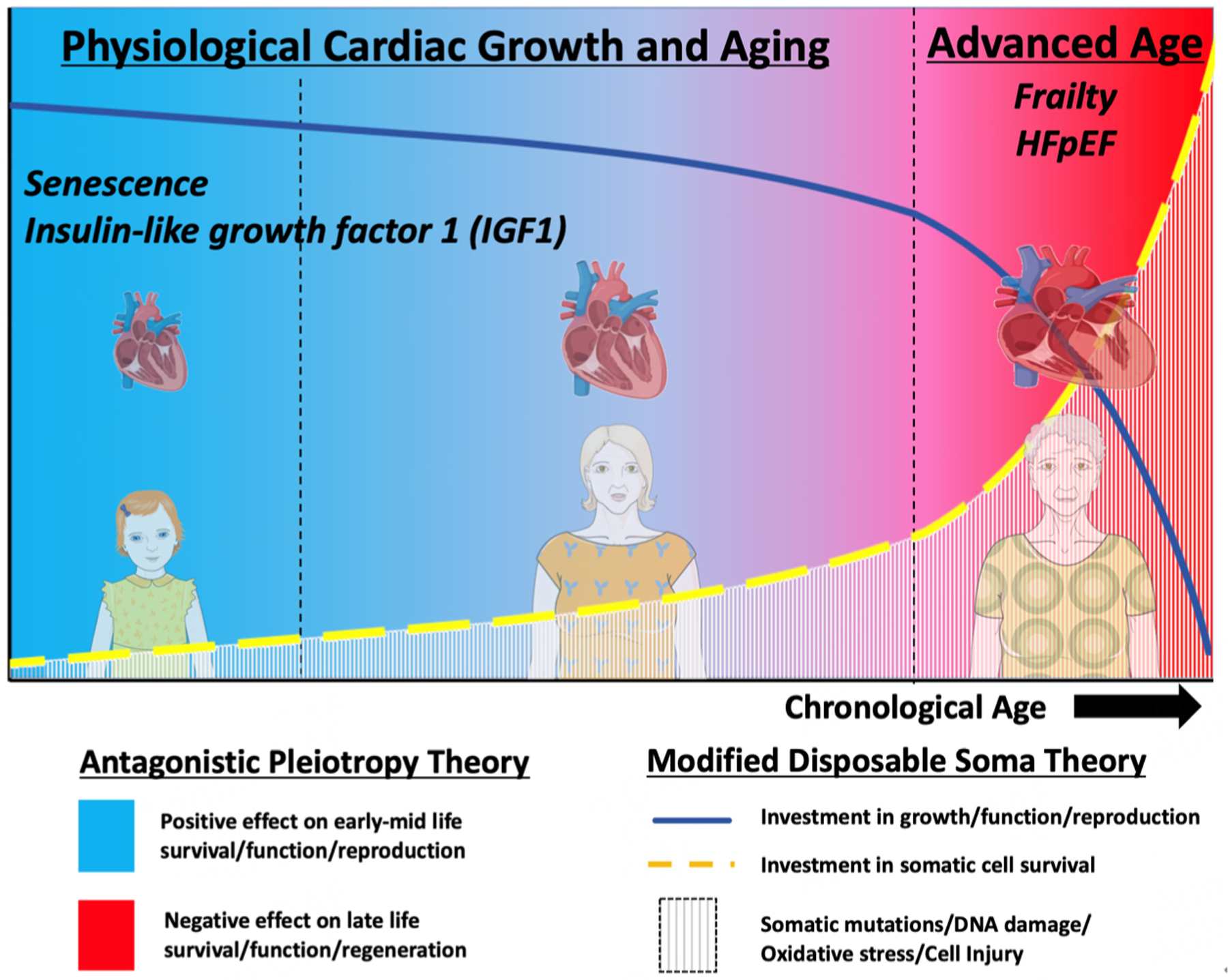
Similar fundamental biology underlying aging and heart failure. The antagonistic pleiotropy theory of aging proposes that evolution strongly selects for traits that maximize early-life survival, growth, and reproduction. However, these same traits can be deleterious in late life and lead to aging and chronic diseases, such as heart failure. Classic examples of this are senescence and IGF1 signaling, which enhance survival and growth in early life, but contribute to adverse cardiac remodeling and dysfunction in late life. The modified disposable soma theory of aging proposes that in early life, organisms will prioritize resources for biological processes that maximize early mid-life survival/growth/function/reproduction (at the expense of maintaining the soma), ultimately leading to accumulating DNA damage and aging. In later life, organisms shift priorities and resource utilization to maintain the soma, even at the expense of function. However, chronic activation of these survival pathways (e.g., senescence, Akt signaling) can become maladaptive, contributing to further adverse remodeling and functional decline in the aging heart. This illustration was partly generated using images adapted from BioRender and Servier Medical Art (https://smart.servier.com). Servier Medical Art is provided by Servier, licensed under a Creative Commons Attribution 4.0 license (https://creativecommons.org/licenses/by/4.0/).

At a fundamental level, the parallels between aging and HF likely stem from similar core biology [Figure 2]. Evolutionary theories of aging, including the theory of antagonistic pleiotropy^[24]^ and the disposable soma theory^[25]^, propose that while evolution favors traits that maximize early survival, growth, and reproduction, these same traits can have deleterious effects in late life. Additionally, as mutations, DNA damage, and oxidative stress accumulate in the soma over time, resource utilization shifts toward pathways to maintain its survival, often at the expense of function. The “healthy” aged heart is no exception. Processes critical for optimal cardiac function, such as fatty acid metabolism, Ca^2+^ cycling (e.g., SERCA2a), and β-adrenergic sensitivity, are all downregulated in the aged heart^[21,23]^, while survival pathways, such as senescence and Akt signaling, are activated. As these cell survival pathways become chronically activated, they become maladaptive and contribute to adverse remodeling and functional impairments in the aged heart^[26–28]^, similar to the core pathophysiology underlying HF^[6,29]^. Ultimately, the unintended consequences of these early life selection forces and the compensatory adaptations that occur later in life contribute not only to the progressive functional decline associated with organismal aging, but also to the development of chronic diseases, such as HF.

Evidence supporting this conceptual framework in both aging and HF is seen in the hallmarks of aging^[12]^. A quintessential example is senescence, which is a process of irreversible cell cycle arrest that promotes cell survival in response to injury and stress. While senescence protects from cancer and enhances survival/reproductive potential in early-mid life, its persistence in late life contributes to the inflammation and multi-organ system failure, including HF, that occurs with aging^[28,30]^. Anabolic insulin-like growth factor 1 (IGF1) signaling, the first longevity pathway discovered^[31,32]^, provides additional evidence of how aging and HF pathophysiology follow the principles of antagonistic pleiotropy. In humans, higher circulating levels of IGF1 are associated with lower disease risk in youth, but correlate with increased risk for incident disease and death in older adults^[33]^. Indeed, across species, reduced IGF1 receptor (IGF1R) signaling enhances longevity^[34]^. In HF, although early studies found that circulating IGF1 was inversely associated with HF risk in older adults^[35]^, more recent work has elegantly shown that when IGF1R signaling is directly modulated in the heart, it displays a more similar pattern to aging, in which IGF1R activation improves cardiac function in young animals, but promotes heart failure in older ones^[36,37]^. Ultimately, in accordance with the Geroscience hypothesis, understanding this shared (patho)biology between aging and HF could identify novel therapeutic targets for both.

### A human-first approach to translating aging research in heart failure

The integration of Geroscience into cardiovascular research^[14,38]^, combined with the rapidly growing number of interventions that can potentially “reverse” aging, has led to an explosion of biological aging mechanisms with therapeutic implications in HF^[12,22]^. However, it is important to recognize that the success rate of a drug target identified in a preclinical study reaching phase II/III clinic trials is extremely low, especially for cardiovascular diseases^[39,40]^. The reasons for this are multifold^[40]^, involving the high costs of funding large-scale clinical trials and prohibitive safety issues with many experimental therapeutics, as well as intrinsic differences in the biology governing aging and HF between humans and experimental animal models. For example, evolutionary selection pressures likely differ between short- versus long-lived species; thus, genetic drivers of lifespan and healthspan in a worm that lives for 2-3 weeks may be fundamentally different than those for humans. Similarly, most preclinical models of HF use acute cardiac injury or stress in young animals, which may not fully reflect the chronic pathophysiology of human HF or the relevance to aging in older adults with HF^[41]^.

While numerous strategies have been proposed to overcome the “valley of death” between preclinical research and clinical drug development, a common theme has been an increasing emphasis on human biology in initial target discovery^[40,42,43]^. Over the past few decades, remarkable advancements in -omics technology (e.g., genomics, transcriptomics, proteomics, metabolomics) have enabled this human-first approach, providing insights into the shared biology of human aging and HF. Notably, while this approach has revolutionized drug development in cancer, the same has not been true in aging or HF. This is not only due to their complex polygenic nature, but also the heterogeneous systemic (patho)physiology of aging and HF that often requires rigorous assessment in animal models before making the leap to patients^[41,44]^.

Here, we start by reviewing recent -omics studies in human aging and HF to demonstrate how this human-first approach can be used to (1) gain insights into the shared biology between aging and HF; and (2) prioritize those mechanisms of aging biology with greater potential for therapeutic development in HF. The breadth of omics data in aging and HF is beyond the scope of any single review. Here, we focus on genomics and proteomics as a starting point. We discuss how genomics can identify causal genetic determinants underlying the strong association between aging and HF, while proteomics conveys a broader picture of their common (mal)adaptative pathophysiology. We conclude each section by providing a detailed discussion of some of the top candidates from human-based omics discovery, which highlights how this Geroscience approach can lead to effective translation of therapeutics targeting aging biology in HF.

## GENOMICS

### Genome-wide association studies in human aging and heart failure

Genomics is the comprehensive study of the entire genome, including the interaction between genes, their regulation, and their downstream effects in the organism. Monogenic causes of premature aging (e.g., progeria) or cardiomyopathy have provided important, albeit relatively specific, insights into the biology of aging and HF. Given the heterogeneity of these complex traits, more recent efforts have utilized genome-wide association studies (GWAS) to better understand their polygenic underpinnings. GWAS involve the genotyping of large populations, usually on the scale of 10^4^-10^6^ individuals, in which statistical associations between millions of germline variants and pre-defined traits of interest (e.g., longevity) are tested. Methods, such as quantitative trait locus (QTL) analysis, colocalization, and Mendelian randomization (MR), are then utilized to assess putative functional effects or infer causality of candidate variants.

Extreme age cutoffs, parental lifespan, or healthspan have been used to define the longevity trait in genomics. Heritability of human longevity is estimated at ∼16% or less^[45]^, and to date, GWAS have identified > 100 loci associated with human longevity [Table 1]^[46–51]^. Interestingly, offspring of longer-lived parents have ∼14% lower risk of developing HF and lower genetic risk scores for HF risk factors^[52]^, suggesting there may be common genetic drivers in aging and HF. Similar to longevity, the heterogeneity of HF has made GWAS challenging. However, collaborative efforts across multiple consortiums are leading to the identification of multiple loci associated with HF or its endophenotypes (e.g., HF risk factors, quantitative imaging traits, *etc*.) [Table 1]^[53–57]^.

The overlap in genetic variants associated with both longevity and HF is not extensive. However, two shared between these complex traits are notably loci related to senescence, including *CDKN2A/B* (p14/p15/p16/ARF cluster) and the regulatory non-coding RNA *CDKN2B-AS1* (also known as ANRIL). While variants near the *CDKN1A* gene (which encodes the senescence protein p21) have not reached genome-wide significance in human longevity studies, they have repeatedly emerged in HF GWAS with MR analysis inferring causality in HF and LVEF endophenotypes^[54,56,57]^. As previously alluded to, senescence represents a quintessential example of how evolutionary theories explain aging [Figure 2]. The association of these loci in both human longevity and HF points to senescence as a potential causal process of aging biology in HF, which will be further discussed in the upcoming sections.

### Somatic mutations in human aging and heart failure

While GWAS identify associations between germline variants (i.e., what we are born with) and traits of interest, most of the body’s cells (collectively referred to as the soma) will also accumulate DNA mutations throughout their lifetime. Most somatic mutations have little or no effect on the cell, but their aggregate accumulation increases the risk of deleterious functional mutations, which is the premise of the classical DNA damage theory of aging^[58]^. Recent progress in next-generation sequencing has led to exciting new insights into the genomic mosaicism that occurs with aging, supporting a functional role of somatic mutations in chronic disease pathogenesis^[59]^.

Our knowledge of how age-associated somatic mutations contribute to HF pathobiology is rapidly evolving. Elegant work by Choudhury *et al*. recently showed that human cardiomyocytes accumulate ∼124 somatic mutations per year, a rate 3 times faster than other nondividing cells, such as neurons^[60]^. While this work provides the most rigorous proof that somatic mutation accumulates in the aging human heart, the functional relevance of these findings remains to be determined. Although not specific to the heart, recent work in clonal hematopoiesis of indeterminate potential (CHIP) provides insights into some of this shared pathobiology between aging and HF. CHIP refers to the clonal expansion of hematopoietic-lineage cells, which occurs due to somatic mutations in key epigenetic regulators (e.g., *DNMT3A, TET2, ASXL1, JAK2*). While CHIP is exceedingly rare in young adults, it increases progressively with chronological age, and is present in nearly 20% of adults greater than 90 years old^[61]^. CHIP is also associated with other hallmarks of biological aging, including epigenetic modifications, telomere shortening, and senescence^[62–65]^; however, whether there is a causal link between these hallmarks and CHIP is unclear. Notably, a series of landmark papers in 2014 and 2017 showed that CHIP is not only strongly associated with, but likely causal, in age-related atherosclerotic cardiovascular diseases^[61,66]^. Subsequent work has now shown similar relationships with HF^[66–69]^. The mechanisms by which CHIP contributes to HF pathophysiology are not fully elucidated, but likely stem from cell-intrinsic proinflammatory processes of these clonal lines, which could explain the inflammatory pathophysiology in aging and HF^[70]^. Emerging evidence from animal models strongly supports this hypothesis, which will be discussed in more detail in the following sections.

**Figure 3.**
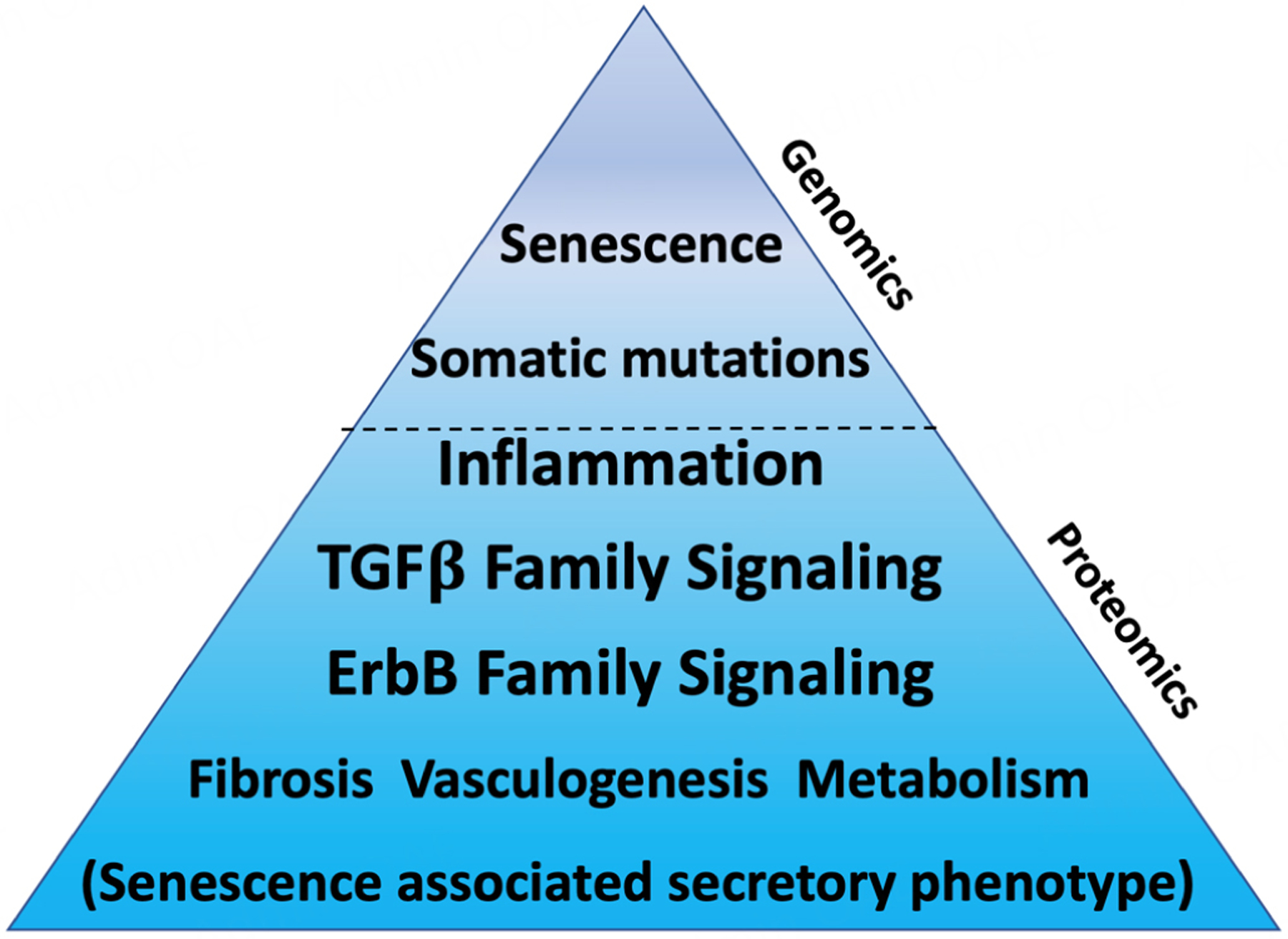
Shared (patho)biology of human aging and heart failure identified from genomics and proteomics. Genomics has identified senescence and age-associated accumulation of somatic mutations/inflammation as central mechanisms of aging biology that contribute to HF pathogenesis. Proteomics provides insights not only into the downstream effects of these two hallmarks of aging in HF pathogenesis, but also into the maladaptive processes in later life that link aging and HF.

In summary, genomics points to common genetic drivers of human aging and HF that are rooted in senescence biology and somatic mutation-driven inflammation [Figure 3]. While epigenomics and transcriptomics, which are not reviewed in this article, provide even greater depth into how these genetic underpinnings of aging contribute to HF pathophysiology, we believe this provides a starting point for prioritizing these hallmarks of aging biology for HF therapeutic development. Next, we review how functional assessments of these candidates in preclinical models have strengthened the rationale for pursuing them in HF.

### Preclinical evidence supporting translation of genomic candidates in heart failure

#### Senescence biology

Cellular senescence is a programmed response to cell stress that occurs with aging and disease^[30]^. The activation of the p53/p21^Cip1^ and p16^Ink4a^/Rb pathways are central to this process, leading to a permanent state of cell cycle arrest that promotes resistance to apoptosis. Senescence is characterized by DNA damage responses, telomere shortening, senescence-associated β-galactosidase (SA-βGal) activity, mitochondrial dysfunction, and metabolic reprogramming. Notably, senescent cells remain metabolically active and develop a senescence-associated secretory phenotype (SASP) characterized by pro-fibrotic and proinflammatory factors that can lead to tissue remodeling and dysfunction^[71–73]^.

In animal models, senescence has been linked to the physiological decline that occurs in organ systems with aging^[71,74,75]^. This includes the aging cardiovascular system, in which senescent cells contribute to adverse cardiac and vascular remodeling, endothelial dysfunction, and atherosclerosis^[28,72,76,77]^. In the aging heart, senescent cardiomyocytes produce a SASP profile that contributes to age-associated cardiac hypertrophy, fibrosis, systolic and diastolic dysfunction, and loss of regenerative potential^[28,75,78]^. The drivers of senescence in aged cardiomyocytes are multifold, including DNA damage, telomere damage, oxidative stress, NFkB signaling, and TGFβ signaling^[28,79,80]^. Senescence not only increases in the aged heart, but has also been implicated in multiple cell lineages and HF syndromes. Endothelial senescence contributes to HFpEF phenotypes, including left ventricular hypertrophy, diastolic dysfunction, and exercise intolerance, in the obese senescence-accelerated mouse^[81]^. Cardiomyocyte senescence contributes to maladaptive remodeling in the ischemia-reperfusion mouse model^[82]^. Cardiac fibroblast senescence increases in the transverse aortic constriction (TAC) model and is associated with perivascular fibrosis^[83]^. Recently, our group also found that placental-derived SASP contributes to cardiac dysfunction and adverse myocardial remodeling in a mouse model of peripartum cardiomyopathy (PPCM), an idiopathic form of acute HFrEF that occurs with pregnancy^[84]^.

Conversely, decreasing senescence has been shown to improve cardiac remodeling and function in both animal models of aging and HF. In aged mice, removing senescent cells, through either pharmacologic senolytics or genetic clearance (e.g., p16-INK-ATTAC), reduces age-associated cardiac hypertrophy and myocardial fibrosis, and improves diastolic function and regenerative potential of the heart^[28,75,78]^. Senolytic approaches have also been shown to improve LV remodeling and systolic function in MI^[82,85,86]^, afterload^[87]^, chemotherapy cardiotoxicity^[88]^, and PPCM^[84]^ models of HF.

The pharmacologic senolytics used in the aforementioned preclinical studies include navitoclax (ABT-263), dasatinib and quercetin (D&Q), and fisetin. Navitoclax is an inhibitor of several Bcl‐2 family proteins through which it selectively induces apoptosis of senescent cells. Dasatinib is a Src/tyrosine kinase inhibitor, and quercetin a natural flavonoid that binds to Bcl-2 and modulates transcription factors, cell cycle proteins, pro- and anti-apoptotic proteins, growth factors, and protein kinases. Fisetin is a plant-based flavonoid with senolytic properties that are mediated through sirtuin activation, NF-kB deactivation, glutathione upregulation, and Bcl-xl inhibition^[89]^. While senolytics are entering early phase clinical trials for various indications, including pulmonary fibrosis^[90]^, Alzheimer’s disease^[91]^, and aging/frailty^[14,92]^, to date, there has been no clinical trial of senolytics in HF. However, senomorphs, or drugs which do not directly kill senescent cells, but target senescence-related pathways, including SASP, are being explored in HF. One example is metformin, which is a synthetic biguanide used to treat diabetes. Although metformin likely has pleiotropic effects in HF, evidence in animal models suggests that some of its benefits are derived from inhibiting SASP^[93–98]^. Post-hoc analyses of clinical trials suggest that metformin potentially lowers mortality and HF hospitalizations in both HFrEF and HFpEF^[99–101]^, and two clinical trials (NCT03629340, NCT05093959) are now testing metformin in HFpEF.

#### Clonal hematopoiesis and inflammation

Animal models have also been used to gain deeper mechanistic insights into how CHIP contributes to cardiac aging and HF. Although spontaneous mutations in human CHIP-associated genes (*TET2, JAK2, DNMT3A*) are not common in healthy aged mice, likely due to their shorter lifespan^[102]^, various strategies to mimic clonal hematopoiesis in mice have been established. Bone marrow transfer approaches, based on competitive transplantation of mutant bone marrow cells or myeloid ablation of driver genes, have been utilized to model CHIP in rodents.

Using these techniques, a series of studies by the Walsh group has provided some of the most compelling preclinical evidence supporting the causal role of CHIP in HF pathobiology. In established murine HF models, including TAC, LAD ligation, and Ang II infusion, *Tet2*, *Jak2*, and *Dnmt3a* deficiency all worsened cardiac remodeling, inflammation, and dysfunction, which were rescued by targeted NLRP3 inflammasome inhibition with the small molecule MCC950^[103–105]^. Notably, a similar mechanistic link between CHIP mutations and the NLRP3 inflammasome occurs in the low-density lipoprotein receptor-deficient (Ldlr^-/-^) mouse model of atherosclerotic cardiovascular disease^[106]^. Similar effects of NLRP3 inhibition have also been reported in HFpEF models, in which MCC950 improves adverse cardiac and pulmonary artery remodeling, diastolic function, and overall exercise capacity, suggesting that the NLRP3-IL1β axis could be a potential therapeutic target for a broad range of HF^[107]^.

While the aforementioned studies prove that clonal hematopoiesis is sufficient to exacerbate cardiac dysfunction in models of cardiac injury and stress, less is known about whether CHIP contributes to physiological cardiac aging. As mentioned, aged mice do not exhibit a high frequency of mutations in human CHIP-associated genes^[102]^. However, mice receiving Tet2-deficient bone marrow transplantation display accelerated cardiac aging with increased cardiac hypertrophy, fibrosis, inflammation, and dysfunction compared to age-matched controls^[108]^. Interestingly, in these mice, Tet2 deficiency also exacerbated age-related cardiometabolic phenotypes^[109]^, which are common in older adults with HFpEF^[110]^.

Whether CHIP pathobiology can be directly targeted as a therapy for HF is an intriguing hypothesis. Inhibitors of JAK1/2 (e.g., ruxolitinib) and TET2 (e.g., azacytidine) have been developed for oncologic treatments and, interestingly, have been shown to improve cardiac arrhythmia and failure phenotypes in murine models^[111,112]^. Notably, the cardiac effects of ruxolitinib are mediated through inhibition of calmodulin-dependent protein kinase II (CaMKII), which is also a mechanism by which CHIP increases atrial fibrillation propensity in mice^[113]^. However, the molecular mechanisms by which these cancer therapeutics modulate cardiac function are still largely unknown. Therefore, whether these drugs can be safely and effectively repurposed for HF is unclear. Of note, clinical studies have not identified any significant increase in major cardiovascular events with JAK inhibitors, but there is a potential signal for cardiotoxicity with TET2 inhibitors^[114–116]^.

Since the mechanism linking CHIP to cardiac aging and HF is largely mediated through the NLRP3-ILβ axis, directly targeting it could be an intermediary approach. The 2017 landmark study, Canakinumab Anti-inflammatory Thrombosis Outcome Study (CANTOS), showed that neutralizing IL1β led to a 15% reduction in major adverse cardiovascular events in patients with previous myocardial infarction and high CRP^[117]^. Interestingly, exploratory analyses found that canakinumab not only led to a dose-dependent reduction in HF hospitalizations and mortality^[118]^, but targeted deep sequencing also revealed that subjects with TET2 CHIP had a greater response to canakinumab than those without it (62% *vs*. 7% reduction in MACE)^[119]^. While these data support the CHIP-IL1β mechanism, prospective studies of the IL1 inhibitor Anakinra have shown mixed effects in HF. While Anakinra lowered NTproBNP and improved exercise capacity at early time points, it did not lead to improved HF outcomes at later time points, despite reductions in CRP^[120]^. Directly targeting the NLRP3 inflammasome may be another approach. Fibrates, which are peroxisome proliferator-activated receptor alpha (PPAR) activators used to lower cholesterol, have been associated with reduced HF hospitalizations^[121]^. Interestingly, they have also been shown to inhibit the NLRP3 inflammasome and improve cardiac inflammation and function in a steatohepatitis-associated cardiomyopathy model^[122]^. The safety of novel NLRP3 inhibitors is now be testing in humans (NCT06336005), which could pave the way for this approach. Ultimately, understanding the pathobiology of CHIP in age-related cardiovascular disease, and identifying those who might benefit the most from CHIP-targeted therapeutics, are important to developing therapeutics that target the inflammaging that accompanies somatic mutations.

## PROTEOMICS

### Proteomics of human aging and heart failure

Proteomics is the comprehensive study of all proteins in a given biological matrix. Unlike one’s germline DNA, which is more or less fixed, proteins are downstream effectors of genes that are highly dynamic, both in terms of their expression levels and post-translational modifications. While this introduces various challenges in interpreting proteomic data, the dynamic nature of the proteome is potentially better aligned with the temporal physiologic and pathologic changes that occur with aging and disease. The incorporation of genetic instruments (e.g., pQTL, MR) into proteomic analyses can help infer causal versus (mal)adaptive compensatory roles of proteins associated with complex traits and can prioritize those with the highest druggable potential^[123,124]^. Notably, proteins currently represent the most common target in clinical biomarker and drug development.

In the past decade, dramatic advancements in high-throughput, multiplex proteomic platforms, including those based on mass spectrometry, modified aptamers (SOMAscan), and proximity extension assays (Olink) now enable simultaneous measurement of thousands of proteins (Olink ∼5,400, SOMAscan ∼11,000) with relatively small quantities (< 100 μL) of biospecimen. Paired with the extensive biobanking and phenotyping efforts of large population cohorts (e.g., UK Biobank), these commercial-based platforms have led to multiple large-scale proteomic studies in both aging and HF. Here, we focus on blood-based (plasma/serum) proteomic studies, but point out that these studies are not tissue-specific (e.g., heart), nor do they address the effects of post-translational modifications or the differences in platforms used^[125,126]^. Despite these limitations, blood-based proteomics has provided valuable insights into the shared biology of human aging and HF.

In the context of aging, while early studies adopting these technologies primarily focused on proteomic signatures associated with chronological age^[127–130]^, more recent efforts have shifted toward using proteomic profiling to gain mechanistic insights into the clinical phenotypes (e.g., longevity, frailty)^[131–135]^ and intrinsic variability of biological aging^[129,136,137]^ [Table 2]. Despite differences in the cohorts, platforms, and aging phenotypes (i.e., chronologic age *vs*. longevity *vs*. frailty), there has been notable consistency in the proteomic signatures identified across these studies. In particular, aging phenotypes are consistently associated with proteins of TGFβ family signaling (FSTL3, MIC-1/GDF15, GDF11/8, TAGL, LEFTY2), ErbB family signaling (ERBB1, FBLN3), inflammation (sTNFR, TREM1, HAVCR2, a2-microglobulin, WFDC), insulin/growth signaling (IGFBP2, IGFBP6, IGFBP7), fibrosis (TIMP1, TSP2, MMPs), vasculogenesis (ANGPT2, SVEP1, VEGF), and metabolism (FABP3, FABP4). Many of these proteins are part of the SASP^[73,138,139]^, suggesting that some of the proteomic signatures of aging may reflect downstream effects of primary senescence biology. Notably, despite these studies excluding subjects with HF, NTproBNP, a clinical biomarker of HF, has consistently emerged as one of the most highly associated proteins with chronologic age, longevity, and frailty. This suggests that either HF is not well adjudicated in these studies, which is unlikely given the consistency of this signal in different cohorts, or current methods are not sufficient to adequately control for the strength of shared biology between aging and HF.

In favor of the latter hypothesis is the striking observation that even after adjusting for age, the majority of circulating proteins associated with incident HF are similar to those associated with aging [Table 2]^[140–144]^. Indeed, a recent large-scale plasma proteomics study of nearly 15,000 subjects identified a similar proteomic signature for incident HF and frailty that largely mirrored circulating proteins implicated in independent aging and HF studies. Of these, FSTL3/GDF15 (TGFβ family signaling), FBLN3 (ErbB family signaling), and TREM1/WFDC1 (inflammation) emerged as potential causal effectors for both frailty and HF (or HF endophenotypes)^[145]^.

In summary, the similar proteomic signatures associated with aging and HF, despite controlling for each other, highlight how strong the systemic biology is between these entities. In many ways, the signatures could reflect downstream effects of senescence and somatic mutation-induced inflammation identified at the genomic level. However, we propose that it likely also reflects the maladaptive compensatory mechanisms that underlie aging and HF (patho)physiology [Figure 3]. Next, we discuss the two candidate processes that exhibit the most consistent positive and negative associations with aging and HF.

### Preclinical evidence supporting translation of proteomic candidates in heart failure

#### Transforming growth factor-β superfamily signaling

While many circulating proteins are associated with both aging and HF, the most highly associated with both have consistently been FSTL3 and GDF15 [Table 2], which are key members of the TGFβ family. In humans, the TGFβ family consists of more than 30 ligands, including TGFβs, activins, lefty, anti-Müllerian hormone, bone morphogenetic proteins (BMPs), and growth differentiation factors (GDFs)^[146]^. These ligands interact with a family of 13 receptors, including 7 type I (ALK1-7), 5 type II serine/threonine kinase receptors (TGFBR2, BMPR2, ActRIIA, ActRIIB, AMHR2), and 1 type III (TGFBR3), which signal through canonical (Smad 2/3 or Smad 1/5/8) or non-canonical (MAPK, JNK/p38, RhoA, PI3K/AKT) pathways. The TGFβ family is involved in a myriad of cellular processes, including proliferation, growth, senescence, survival, inflammation, and fibrosis, which are highly context-dependent and tightly regulated by specific ligand-receptor interactions and multiple inhibitors^[147]^.

FSTL3 is a secreted glycoprotein that neutralizes circulating activins, TGFβs, GDF11, and GDF8. It is a downstream target of Smad 2/3 signaling and functions as a negative feedback loop for ActRII and TGFBR2 receptor signaling. Thus, increased circulating FSTL3 can be interpreted as an indicator of increased signaling through these receptors after binding their cognate ligands. Notably, all four ligands have been implicated in cardiac aging and HF, with the first three also being components of the SASP^[148]^. GDF11 and activins have recently garnered significant attention in cardiac aging and HF, and thus, our discussion will focus on these ligands in addition to GDF15.

GDF11’s role in cardiac aging and HF emerged from a heterochronic parabiosis study in mice, in which it was identified as a potential rejuvenating factor that reverses age-related cardiac hypertrophy, sarcopenia, and impaired neurogenesis^[149–151]^. Follow-up work, however, identified specificity issues with the aptamer used to identify GDF11, suggesting that it also binds the closely homologous GDF8, which was likely responsible for the anti-hypertrophic effects observed in these original studies^[152,153]^. A series of studies have confirmed that GDF11 does reverse cardiac hypertrophy, but at higher doses, it becomes deleterious, causing severe cachexia and increased mortality^[154,155]^. The effects of GDF11 on cardiac function are not clear with studies showing that it both worsens and improves cardiac function in animal models of HF^[156–159]^. In humans, while GDF11 was initially thought to decline with aging, highly specific LC-MS/MS have found that circulating GDF11 levels do not change with age, nor do they relate to increased HF risk in older adults^[153,160]^. However, consistent with plasma/serum proteomics studies [Table 2], LC-MS/MS assays have confirmed that FSTL3 is strongly associated with HF in older adults, particularly HFpEF^[160]^. Based on the mixed preclinical and clinical evidence, GDF11 is not currently being tested as a therapeutic for HF. If it is to be pursued in the future, careful dose optimization and monitoring will be required to ensure safety with its catabolic effects.

Activins, specifically activin A, also increase FSTL3 expression and have been proposed to be one of the major TGFβ family ligands responsible for the increased circulating FSTL3 signal observed in human aging and HF^[161]^. Circulating activin A consistently increases with chronologic age in both sexes, particularly in the last decades of life^[162]^, and in those with frailty^[163]^. Similar findings are also observed in mice, where circulating activin A levels increase with age and correspond to age-associated increases in cardiac FSTL3 expression^[161]^. Moreover, in both naturally aged mice and progeria models (Ercc1 Δ/−), inhibition of activin A, through either antibodies against its receptors (ActRII) or soluble ActRII ligand traps, improves age-associated cardiac dysfunction and remodeling^[161,164]^. These findings extend to HF in both humans and rodent models. Circulating activin A levels correlate with worse HF severity in humans^[84,161,165]^. Conversely, inhibition of activin A or ActRII improves cardiac function and remodeling in multiple animal models of HF, including TAC, LAD ligation, aging, cancer, dilated cardiomyopathy, and PPCM^[84,161,166–168]^. The mechanisms underlying the benefits of activin-A/ActRII inhibition in HF are multifold, encompassing effects on proteostasis, Ca^2+^ handling, fibrosis, energetics, metabolism, and senescence. It is important to note that while chronic increases in activin A are sufficient to induce cardiac dysfunction^[161,169]^, acute increases have been shown to improve cardiomyocyte survival in ischemic injury^[170]^. While this may seem contradictory, it likely follows the paradigms underlying aging and HF, in which chronic activation of survival pathways becomes maladaptive. Targeting activin-A/ActRII biology in HF follows the recent success of inhibiting it in pulmonary artery hypertension (PAH)^[171,172]^. Sotatercept, an ActRIIA-Fc that has been FDA-approved for PAH and has been shown to have anti-inflammatory and anti-proliferative effects on vascular cells^[173]^, is now being tested in HFpEF (NCT04945460), while bimagrumab, an antibody to ActRII which has beneficial metabolic remodeling and antifibrotic effects^[174,175]^, is being tested in obesity/cardiometabolic disease (NCT05616013).

Unlike GDF11 and activin A, growth differentiation factor 15 (GDF15) is a distant member of the TGFβ superfamily that does not bind to any of its 13 receptors, but rather the orphan receptor, GDNF-family receptor a-like (GFRAL)^[176]^. GDF15 is expressed at very low levels under physiological conditions, but is markedly upregulated following injury. As highlighted in the proteomics of human aging and HF [Table 2], GDF15 is strongly associated with aging, frailty, and HF, and is a predictor of all-cause mortality^[177–179]^. However, it remains unclear whether GDF15 is just a biomarker of cellular injury and inflammation in these contexts or if it plays a functional role that can be targeted for therapeutic development. Multiple aspects of GDF15’s biology suggest that similar to activins and senescence, it likely follows the same evolutionary principles that dictate aging and disease^[180]^. GDF15 exhibits potent anti-inflammatory and catabolic effects^[181,182]^, and has been shown to increase longevity in mice^[182]^. In the cardiovascular system, GDF15 increases endothelial senescence/dysfunction and cardiac fibrosis^[183–185]^, but in HF models, induced by LPS, TAC, or ischemia/reperfusion, GDF15 is generally cardioprotective^[181,186–188]^. However, emerging evidence suggests that inhibiting GDF15 could conversely improve cardiac cachexia and HF progression by blocking atrophy pathways induced by GDF15^[189–192]^. While the catabolic effects of GDF15 largely mirror that of GDF11, a notable difference between these TGFβ family members is that GDF15 levels are consistently and highly increased in aging and HF, while GDF11 levels are generally unchanged in these contexts. This makes the former perhaps more relevant to aging and HF pathobiology. While no clinical trials targeting GDF15 are currently underway in HF, both antagonistic and agonist tools are being explored for other clinical indications. Visugromab, a GDF15 neutralizing antibody, is being tested in patients with advanced stages of cancer (NCT06059547, NCT04725474), while LY3463251, a long-acting GDF15 receptor agonist, is being investigated as an anti-obesity treatment, though dose-dependent nausea and emesis may be a limiting factor with the latter^[193]^.

#### ErbB family signaling

While proteins implicated in TGFβ family signaling are consistently among the most positively associated with human aging and HF, the only protein that is negatively associated with both is ErbB1. The ErbB proteins represent a family of four closely related tyrosine kinase receptors, which include ErbB1 (also known as epidermal growth factor receptor EGFR), ErbB2, ErbB3, and ErbB4. The ErbB receptors bind a large family of ligands, including EGF, neuregulins (NRGs) 1-4, TGF, amphiregulin, betacellulin, epiregulin, and epigen. The ErbB family of ligands-receptors are essential in cardiac development, but have also been implicated in disease, including HF^[194,195]^.

NRGs, in particular, have gained attention in HF research, given their therapeutic potential. NRG-1 is the most abundantly expressed member of this family in the heart. There are two variants of NRG1, α and β, with NRG1β being the more active isoform. NRG1 binds directly to ErbB3 and ErbB4, which in coordination with the orphan receptor ErbB2, form homo- and hetero-dimers. Signaling pathways through ErbB include PI3K-AKT, ERK-1/2, and Hippo-YAP, which regulate cardiomyocyte survival, growth, and proliferation^[196–198]^. NRG1 also plays a central role in the development and function of non-cardiomyocyte lineages, including neurons, endothelial cells, fibroblasts, and immune cells^[199–204]^, and serves as a central regulator of the intercellular crosstalk and interplay between cardiomyocytes, the microvasculature, and conduction system in the heart.

While NRG1/ErbB signaling has been studied extensively in early cardiac development, its role in cardiac aging is less well understood. Sustained higher circulating levels of NRG1 have been found in long-lived species such as the naked mole rat^[205]^, suggesting that it may play a role in promoting survival and longevity. NRG has also been shown to attenuate vascular senescence^[206]^, and in the aged heart, downregulation of NRG/ErbB signaling is associated with mitochondrial dysfunction and cardiomyocyte apoptosis^[207]^. As a mediator of the adaptive response to physiological stress, NRG1 also contributes to physiological cardiac hypertrophy in both pregnancy and exercise^[208,209]^.

The potential therapeutic role of NRG/ErbB signaling in HF emerged from clinical safety data indicating an increased risk of HF and systolic dysfunction in patients with breast cancer being treated with trastuzumab (a humanized monoclonal antibody targeting ErbB2)^[210,211]^. This observation was supported by evidence from transgenic animal models, which showed that cardiac or cardiomyocyte-specific genetic ablation of ErbB2 or ErbB4 was sufficient to induce dilated cardiomyopathy^[212–214]^. NRG1/ErbB signaling was subsequently shown to be reduced in both human and experimental HF models^[215,216]^, and activating cardiac ErbB signaling via recombinant NRG1 improved cardiac function and survival in multiple animal models of HF^[217]^. The mechanisms by which NRG1/ErbB improves cardiac function are multifold, including its effects on cardiomyocyte Ca^2+^ signaling, sarcomere function, hypertrophy, metabolism, survival, and regeneration, as well as its impact on angiogenesis and fibrosis^[196,197,202,204,206,218,219]^.

Based on the collective preclinical and clinical evidence, multiple clinical trials testing the safety and efficacy of recombinant NRG1 have been implemented in HF^[220,221]^. In patients with chronic HF, short-term treatment of rhNRG1 (Neucardin) improved hemodynamics, LVEF, and some metrics of adverse LV remodeling. A larger full-length recombinant NRG-1B3 (cimaglermin) has also been tested in chronic HFrEF, and has similarly shown improvements in LVEF, but dose-limiting toxicity with liver injury^[222]^. Studies in rodent and swine models suggest that the cardiac benefits of NRG-1B3 are likely mediated through improvements in cardiomyocyte mitochondrial function and reduced cardiac fibroblast activation^[223,224]^. Lastly, in attempts to avoid potential cancer-promoting effects of ErbB2 activation, engineered ErbB3-NRG1 antibody constructs (JK07) have been designed to bias signaling toward ErbB4. An early phase I study shows this approach is safe, with signals suggesting it increases LVEF in patients with chronic HFrEF^[225]^. Similar to NRG-1B3, preclinical data from rodents and non-human primate HF models suggest that JK07 reduces myocardial fibrosis and improves diastolic function^[226]^. Phase II/III studies testing the efficacy of recombinant NRG1 (NCT03388593) and JK07 (NCT06369298) in chronic HFrEF are currently underway.

## SUMMARY

Advancements in our understanding of the biology of aging are changing the perception of age in HF. The ability to modify biological aging and effectively alter HF pathophysiology offers the exciting possibility that aging itself could represent a new area for therapeutic target discovery for HF. While Geroscience is gaining traction in cardiovascular disease research^[14,38]^, ensuring its success and sustainability in the field will inevitably require that certain metrics be achieved in the coming years. Given that the overarching mission of Geroscience is to tackle chronic diseases by targeting aging biology, it is imperative that the investment into this emerging area of interdisciplinary research fulfills its promise of developing effective gerotherapeutics. HF, with its overwhelming prevalence in the elderly population and its markedly similar (patho)biology and physiology to aging, perhaps represents the ideal challenge for Geroscience. As highlighted in this review, prioritizing those targets with strong evidence based on the shared biology of human aging and HF will ultimately enhance the translational success of this Geroscience approach and hopefully change the trajectory of HF in older adults.

## Figures and Tables

**Table 1.** Genome-wide association studies in human aging and heart failure. This table summarizes the variants that are associated with aging traits (longevity, parental lifespan, healthspan) or incident HF (and HF endophenotypes). Bold indicates gene loci associated with both aging and HF traits

Study	Population	Size	Associated genes
**Longevity**			
Deelen *et al*., 2014^[46]^	Meta-analysis of 14 European ancestry studies	44,368	MTR, RYR2, CSRNP3, PDE5A, MAD2L1, EBF1, EYA1, CYP2C19, TANC2, APOE, TSH22, ZNF217, NTN1, MARCH10, TANC2
Broer *et al*., 2015^[47]^	Cohorts for heart and aging research in genomic epidemiology (CHARGE)	9,793	GRIK2, CADM2, RGS7, SOX6, MBOAT1, PFKM, LIMCH1, FOXO3
Yashin *et al*., 2015^[48]^	Framingham heart study	1,529	TLK2, C1QTNF5, ECHS1, RIMBP2, CYP51A1, WRN, NRDE2
Zeng *et al*., 2016^[49]^	Chinese longitudinal healthy longevity study (CLHLS)	4,477	IL6, ANKRD20A9P, MIR3156-3, AKR1C2, FAM13A, BEND4, EPHA6, ZFYVE28, ASIC2, OLFM4, APOE
Gurinovich *et al*., 2021^[50]^	New England centenarian study; LonGenity	5,169	APOE, SHB, ALDH1B1, FBX015, LRRC3B, APBA2, CCNC, PRDM13, MYYO5B
Deelen *et al*., 2019^[51]^	Meta analyses of 23 studies (18 European, 1 East Asian, 1 African American)	142,203	GRP78, RP1, RDH5, APOE, KALRN, K1F13B, **CDKN2A/B**
**Parental lifespan**			
Pilling *et al*., 2017^[227]^	UK biobank	389,166	**CELSR2**, HLA-DRB1, **LPA**, **CDNK2B-AS1**, **ATXN2**, CHRNA3/4, FURIN, APOE, BEND3, EPHX2, PROX2, MC2R, TNNI3K, USP2-AS1, PSORS1C3, B3GALT3L, MICA/B, TOX, ZW10, SEMA6D, EGLN2, CYP2A6, EXOC3L2, MARK4, C20orf187
Joshi *et al*., 2017^[228]^	CHARGE	606,059	HLA-DQA1/DRB1, **LPA**, CHRNA3/5, APOE
Wright *et al*., 2019^[229]^	AncestryDNA	> 300,000	**CDKN2B-AS1**, WAPL, **LPA**, SRRM3, CHRNA3/5, APOE
Timmers *et al*., 2019^[230]^	UK biobank, LifeGen	1,012,240	MAGI3, KCNK3, HTT, HLA-DQA1, LPA, **CDKN2B-AS1**, **ATXN2**/BRAP, CHRNA3/5, FURIN, HP, LDL3, APOE
**Healthspan + parental lifespan + longevity**			
Timmers *et al*., 2020^[231]^	UKBiobank	1,349,462	**CELSR2**, MAGI3, KCNK3, CASP8, SLC4A7, HTT, LINC02513, DUSP22, HLA-DRB1, FOXO3, **LPA, CDKN2B-AS1**, TCF7L2, TYR, ZW10, FGD6, ATXN2, CHRNA3, FURIN, TOX3, DEF8, LDLR, APOE, NOL4L
**Heart failure**			
Smith *et al*., 2010^[53]^	CHARGE	23,821	USP3, LRIG3, BCHE, EVX1, SNX16, MOBKL2B, CH25H, PRICKLE1, TBC1D4, GNA15, SH3GL2, RPUSD4, TMTC1, BTG1, HLX-AS1, TEX51
Shah *et al*., 2020^[54]^	Heart failure molecular epidemiology for therapeutic targets (HERMES) consortium	977,323	**CELSR2**, PITX2, FAM241A, KLHL3, CDKN1A, **LPA**, **CDKN2B-AS1**, ABO, SURF1, SYNPO2L, AGAP5, BAG3, **ATXN2**, FTO
Levin *et al*., 2022^[55]^	HERMES, eMERGE, Penn Med Biobank, Mt Sinai BioME, geisinger DiscovEHR, FinnGen, global biobank	1,665,481	RP11, NPC1, ZFHX3, **CDKN2B**, PITX2, HDGFL1, FTO, CDKN1A, SURF6, SYNPO2L, CLCNKA, IRAK1BP1, FAM133B, STRN, BAZ1A, SRR, GNPDA2, PMAIP1, TMEM18, GTF2I, HSD17B12, BAG3, HECTD4, **LPA**, SH2B3, DMRTA2, USP36, OR2A2, POM121C, CACNB2, ASXL3, CHMP3, TTC39A, NFIA, KCNIP4, TUBA3C, ORC5, ZNF280A, COX7C
Joseph *et al*., 2022^[56]^	Million veteran program, UK biobank	529,386	SPARP, HSPB7, ZBTB17, **CELSR2**, E2F6, ABHD5, PITX2, CDKN1A, **LPA**, GTF2I, **CDKN2B-AS**, ABO, CAMK2G, BAG3, FTO, NFAT5, SMG6, PNMT, PGAP3, YPEL2, BPTF, MAP3K7CL
Rasooly *et al*., 2023^[57]^	HERMES, million veteran program	1,279,610	HSD17B12, HDGFL1, SPATS2L, TMEM18, SCARB1, CAMK2D, FAF1, GNPDA2, POM121C, ZEB2, RIC8B, FANCL, PRKD1, SLC39A8, HEATR5B, PHIP, SYMPK, SGIP1, **CDKN2B**, PITX2, FTO, **LPA**, CDKN1A, PSRC1, C1orf64, BAG3, ABO, SYYNPO2L, BPTF, KLHL3, BACH1, GTF21, NFAT5, SMG6, ALDH2, MIA3, PNMT, PHACTR1

**Table 2.** Proteomic studies in human aging and heart failure. This table summarizes recent blood-based (plasma or serum) proteomic studies that test associations of circulating proteins with aging (chronological age, longevity, frailty) or incident HF (and HF endophenotypes). Only the top 30 most significant proteins from each study are included. Bold indicates proteins that were inversely associated with the phenotype

Study	Proteomic platform	Discovery cohort	Validation cohort	Protein associations (up to top 30 most significant)
**Chronological age**				
Menni *et al*., (2015)^[127]^	SOMAscan, 1.1 K	TwinsUK (*n* = 206)	AddNeuroMed, Alzheimer’s Research UK/Maudsley MRC Dementia Case Registry (*n* = 677)	CHRDL1, CCDC80, PTN, FSTL3, TIMP1, MMP12, Cystatin C, IGFBP6, ROR1, THBS4, HAVCR2, ADAMTS5, EDA2R
Tanaka *et al*., (2018)^[128]^	SOMAscan, 1.3 K	BLSA, GESTALT (*n* = 240)	NA	MIC-1/GDF15, PTN, ADAMTS5, FSH, SOST, CHRDL1, NTproBNP, FBLN3, MMP12, **Cathepsin V**, FSTL3, b2-microglobulin, hCG, CAPG, sTNFR-I, TIMP1, NBL1, CCD80, Cystatin C, CHIT1, IGFBP2, **RET**, EPHA2, LUM, IL18BP, PPY, LTBP4, IGFBP6, DSC2, SMOC1
Lehallier *et al*., (2019)^[129]^	SOMAscan, (1.3 K, 4.0 K, 5.0 K)	INTERVAL, LonGenity (*n* = 4,263)	VASeattle, PRIN07, PRIN09, GEHA (*n* = 171)	SCARF2, ARFIP2, SOST, PTN, WFDCC2, **AGRP**, MIC-1/GDF15, MXRA8, **IGDCC4**, FSH, **CD93**, **MSMP**, NTRK3, **CHAD**, ADAMTS5, **COL15A1**, MLN, **CDON**, KLK3, **SCG3**, **PTPRD**, **KLK7**, C1QTNF3, DKL1, CTSF, SSVEP1, **DSG2**, **RET**, CCDC80
Sathyan *et al*., (2020)^[130]^	SOMAscan, 5.0 K	LonGenity (*n* = 1,025; *n* = 506 OPEL; *n* = 519 OPUS)	NA	PTN, WISP-2, CHRDL1, TAGL, RSPO1, FBLN3, **ERBB1**, MIC-1/GDF15, SMOC1, HE4, PGD2 synthase, Cystatin C, FSTL3, RNASE1, MSR1, URB, CCD80, **a2-Antiplasmin**, sTREM1, NTproBNP, SREC-II, b2-Microglobulin, ASB9, CDCP1, HPB6, ATS13, TNF sR-II, SVEP1, SAP33, TMEDA, SET
**Longevity**				
Orwoll *et al*., (2020)^[131]^	SOMAscan, 4.0 K	MrOs (*n* = 2,473)	NA	**C9**, **S100A9**, **CD163**, **CRP**, **IGHM**, **C7**, **FCG3A**, **LG3BP**, **NRP1**, **CD166**, **PHLD**, **b2-microglobulin**, **a2-microglobulin**, **MMP2**, **vWF**, **CSF-1R**, **HPRT**, **CFAD**, **CD5L**, **FCGBP**, **IGHG3**, **Prothrombin**, **Cystatin C**, **PTGDS**, **MUC18**
Liu *et al*., (2024)^[134]^	SOMAscan, 5.0 K	CHS (*n* = 3,067)	AGES-Reykjavik (*n* = 4,690)	**MIC-1/GDF15**, **NTproBNP**, ERBB1, **TSP2**, **MMP-12**, Amyloid-like protein 1, CILP2, **Endostatin**, NOTUM, **CD14**, Protein C, **SRSF7**, **TWSG1**, **PXDN**, **b2-Microglobulin**, **RNase 1**, NG36, SET, **Epididymal secretory protein E1**, **EPHAA**, **HE4**, **Angiopoietin-2**, **aTL3**, **sTNFR-II**, **BAGE2**, **TRA2B**, MXRA8, **Cystatin C**, **FBLN3**, **EWS**
**Frailty**				
Sathyan *et al*., (2020)^[232]^	SOMAscan, 5.0 K	LonGenity (*n* = 880)	NA	**ANTR2**, FABP3, FABP4, **ERBB1**, **NELL1**, **MXRA8**, **CA2D3**, Leptin, **DNER**, **PTGD2**, **NCAM-120**, **CSPG3**, Coag Factor IXab, **GDF11/8**, **Contatin-1**, **NG36**, **ADAM22**, **ENPP5**, **HS6ST3**, IL-1Ra, FSTL3, PXDN, HTRA1, **ARFP2**, **ANTR1**, **FPRP**, SSRF7, Coag Factor IV, **NEO1**, **Tenascin-X**
Liu *et al*., (2022)^[134]^	SOMAscan, 4.0 K	CHS (*n* = 2,854)	FHS (*n* = 1,130)	MIC-1/GDF15, PTN, TIMP1, Troponin T, **IL-7Ra**, NET1, PARC, DAN, ANGPT2, FSTL3, URB, CD59, RSPO3, Carbonic anhydrase 3, **CDON**, SMOC1, SHP-2, **RANTES**, Cathepsin D, PHI, Endostatin, eIF-5, **ERBB1**, CRK, Cripto, GSTP1, Ubiquitin, **C1R**, MMP7
Liu *et al*., (2023)^[133]^	SOMAscan, 4.0 K	ARIC (*n* = 3,838 cross-section; *n* = 1,725 longitudinal)	CHS (*n* = 2,570 cross-sectional; *n* = 1,817 longitudinal)	TREM1, FABP3, TAGL, FABP4, CAPG, NBL1, RNASE1, MYL6B, **CBLN4**, HSPB6, HAVCR2, **NDST1**, MIC-1/GDF15, WFDC2, FSTL3, LEFTY2, PCCDHGA12, sTNFR-1, TFF2, VEGFA, PTN, NET1, DNAJB9, SUMF1, Angiopoietin-2, SELH, COL28A1, REG3A, NTproBNP, WFDC1
**Mortality**				
Kuo *et al*., (2024)^[135]^	Olink, 3,072	UKBiobank (*n* = 53,021)	NA	MIC-1/GDF15, WFDC2, NEFL, EDA2R, PLAUR, TFFR, sTNFR-I, MMP12, LTBP2, AREG, HGF, ADM, IGFBP4, VSIG4, TGFA, CEACAM5, PIGR, IL6, FGL1, LAMP3, THBS2, CLEC3B, IFI30, OSM, IL32RA, NTproBNP, CSF1, Angiopoietin2
**Incident Heart Failure**				
Ferreiera *et al*., 2019^[140]^	Olink, CVD II, CVD III, inflammation panels	HOMAGE (*n* = 877)	HOMAGE (*n* = 556)	NTproBNP, TRAILR2, sTNFR-1, GAL-9, GFG23, REN, MIC-1/GDF15, FABP4, SLAMF7, CCL16, **TWEAK**, KIM1, CD4, VSIG2, **PON3**, PLFG, MMP-12, ADM, RARRESS2, CEACAM8, SLAMF1, AGRP, TNFR2, IGFBP7, UPAR, PAR1, PLC, ACE2, IL16, TFF3, OPN, SPON2, ILLR-4AR, TNFRSF14
Nayor *et al*., 2020^[141]^	SOMAscan, 5.0 K	FHS (*n* = 1,913)	HUNT (2,515)	NTproBNP, **ERBB1**, MBL, **TRMC**, **GDF-11/8**, TSP2, NRP1, vWF, **ApoE4**, **ApoE3**, **RGMA**, **NOTCH 1**, **MnSOD**, **RGMC**, IL-1Ra, **NEGR1**, **APO L1**, **ISLLR2**, **VEGFA sR2**, S100A2, TNFa, MIC-1/GDF15, Cathepsin D, **NCAM-120**, **DKK4**, **IgG**, C7, **PEX5**, **BMPR1A**
Girerd *et al*., 2023^[142]^	O-link, CVD II, CVD III, inflammation panels	ARIC (*n* = 500); FHS (*n* = 382); HOMAGE (1,433)	NA	NTproBNP, BNP, 4E-BP1, HGF, Gal-9, TGFA, THBS2, U-PAR (significant in all three cohorts)
Emilsson *et al*., 2023^[143]^	SOMAscan, 5.0 K	AGES-Reykjavik (*n* = 5,457)	CHS (*n* = 3,484)	NTproBNP, MIC-1/GDF15, CTSH, Cystatin C, RNASE1, Angiopoietin-2, GABARAPL1, MMP12, SVEP1, b2-microglobulin, RELT, MFAP2, PXDN, FABP3, TMPO, ATP5J, COL28A1, FABP4, WFDC2, TFF3, TMED10, MMP7, SPON1, PLAUR, ADM, TNNI3, CCL15, COL6A3, PCDHGA10, TAGL, MDM1
Shah *et al*., 2024^[144]^	SOMAscan, 5.0 K Olink, 3,072	ARIC mid-life (*n* = 10,638), ARIC late-life (*n* = 4,483), HUNT (*n* = 3,262)	NA	NTproBNP, SVEP1, WFDCC2, Angiopoietin-2, MIC-1/GDF15, SPON1, THBS2, **CILP2**, **GHR**, FSTL3, TAGL, IGFBP2, APOF, TREM1, FSTL1, TIMP4, C9, ANGPTL3, SLIT2, IGFBP7, sTNFR-1, MFAP4, CCL15, ITIH3, NRP1, CELA1, **ERBB1**, **CACNA2D3**, **CLEC3B**, **PTPRD**, **ATP1B1**
**Incident frailty and heart failure**				
Ramonfaur *et al*., 2024^[145]^	SOMAscan, 5.0 K	ARIC mid-life (*n* = 10,638) ARIC late-life (*n* = 3,908)	CHF (*n* = 3,189)	COLL28A1, COL6A3, WFDC2, PXDN, LEFTY2, TAGL, GABARAP, CST3, sTNFR -I, NBL1, FSTL3, MIC-1/GDF15, TMED10, RNAse1, FBLN3, TWSG1, TREM1, WFDC1
